# The complete mitochondrial genome of *Tringa semipalmata inornata* (Charadriiformes; Scolopacidae)

**DOI:** 10.1080/23802359.2017.1339213

**Published:** 2017-06-13

**Authors:** Guiqi Bi

**Affiliations:** a Key Laboratory of Marine Genetics and Breeding (OUC), Ministry of Education, Qingdao, China;; b College of Marine Life Sciences, Ocean University of China, Qingdao, China

**Keywords:** *Tringa semipalmata*, mitogenome

## Abstract

In this study, we presented the complete mitochondrial genome of *Tringa semipalmata*. The circular genome is 16,603 bp in length and contains 13 protein-coding genes (PCGs), 22 transfer RNA (tRNA) genes, 2 ribosome RNA genes and 1 D-loop control regions. The overall nucleotide composition is as follows: 31.2% A, 25.2% T, 29.9% C and 13.6% G, with a total G + C content of 43.53%. The phylogenetic tree was constructed to validate the taxonomic status of *T. semipalmata*, exhibiting it a close relationship to other *Tringa* species.

Willets (*Tringa semipalmata*), with grey-brown plumage and a long, thick, grey bill, are widespread, migratory New World shorebirds with two recognized subspecies: the Eastern (*T. semipalmata semipalmata*) and Western (*T. semipalmata inornata*) (Martínez-Curci et al. 2014; Oswald et al. 2016). Since the late nineteenth and early twentieth centuries, the willet's population declined sharply due to hunting. Though their population has since increased, they are still considered at risk, especially in the light of continued habitat loss. And *T. Semipalmata is* still listed as Least Concern (LC) on the IUCN Red List of Threatened Species. To date, the information of its complete mitochondrial genome is still limited, which impedes the further investigation of population dynamics. In this study, to gain further insight into the gene content and structure of *Tringa semipalmata* through mt genome, its whole mitochondrial genome was determined.

The specimen (subspecies of *Tringa semipalmata inornata)* was collected from the shore of Wyoming, USA. The muscle tissue and total genomic DNA that was extracted through Animal Tissues Genomic DNA Extraction Kit (Solarbio, Beijing, China) were stored in the sequencing company (BGI Tech, Shenzhen, China). Purified DNA was fragmented and used to construct the 300 bp libraries following the instructions of NEBNext^®^ Ultra™ II DNA Library Prep Kit (NEB, Beijing, China). Whole genomic sequencing was performed by the Illumina HiSeq 2500 Sequencing Platform (Illumina, San Diego, CA). Adapters and low-quality reads were removed using the NGS QC Toolkit (Patel & Jain 2012). The mitochondrial reads from pre-filtered reads were screened out by bowtie2 (Langmead & Salzberg 2012) using other two *Tringa* mitochondrial genomes as references, and then assembly as implemented by SPAdes 3.9.0 (Bankevich et al. 2012). Gaps among contigs were filled by using MITObim V1.9 (Hahn et al. 2013). The determined genome was annotated using the MFannot tool (http://megasun.bch.umontreal.ca/cgi-bin/mfannot/mfannotInterface.pl), combined with manual corrections. tRNAs were annotated by ARWEN Web Server (Laslett & Canbäck 2008).

The complete mt genome of *Tringa semipalmata* is 16,603 bp in length, which showed high similarity with *Tringa* species both in size, structure and gene content (Chen et al. 2016; Cheng et al. 2016). This mitogenome was submitted to GenBank database under accession No. MF036175. The circular genome contains 13 protein-coding genes (PCGs), 22 transfer RNA (tRNA) genes, 2 ribosome RNA genes and 1 D-loop control regions (Figure 1). The overall nucleotide composition is as follows: 31.2% A, 25.2% T, 29.9% C and 13.6% G, with a total G + C content of 43.53%.

**Figure 1.**
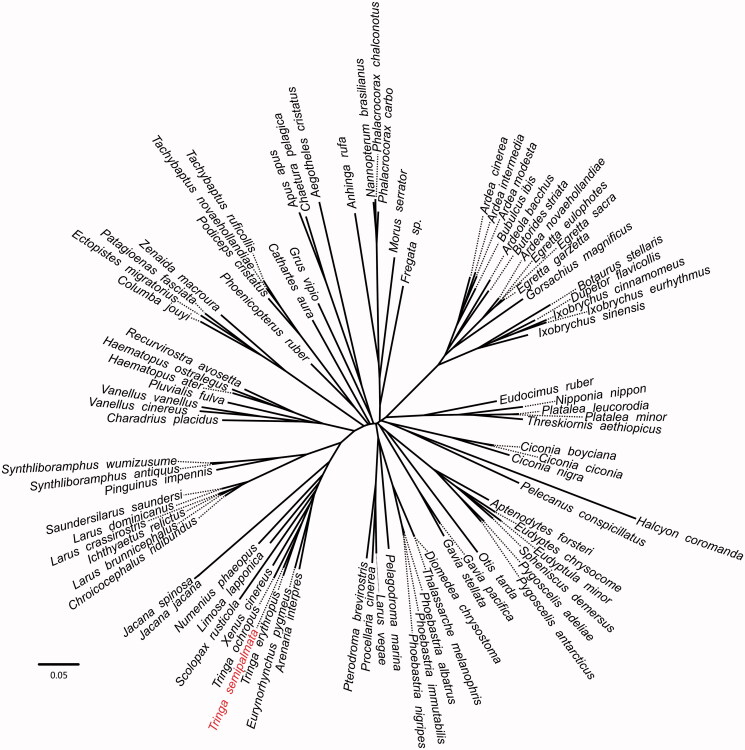
Phylogenetic relationships among 90 bird mt genomes. This tree was drawn without setting of an outgroup. All nodes exhibit above 90% posterior probability (PP) and 85% RAxML supported bootstraps. The length of branch represents the divergence distance.

To validate the phylogenetic position of *Tringa semipalmata*, the genome-wide alignment of 90 birds mitochondrial genomes was constructed by HomBlocks (https://github. com/fenghen360/HomBlocks), resulting in 12,596 characters of each species, which including all PCGs and rRNA genes. The jModelTest 2.0.2 (Darriba et al. 2012) was used to ascertain the best-fit model of nucleotide substitution for sequences with Bayesian information criterion (BIC). Bayesian analysis (BI) and maximum likelihood (ML) were used to reconstruct the phylogenetic trees. Bayesian phylogenetic analysis was conducted with MrBayes 3.2.5 (Ronquist et al. 2012) based on the most appropriate model. Four Markov chains were run for 10,000,000 generations to estimate the posterior probability (PP) distribution (sampling 1 tree with 100 replicates for each run). After discarding the first 2000 trees as burn-in that was referred to log likelihood values, 50% majority-rule consensus trees were estimated for the remaining trees. ML analysis was performed using RAxML (Stamatakis 2014) with the GTR + G + I model. Bootstrap values were calculated using 1000 replicates to assess node support. Phylogenetic relationships obtained with the maximum likelihood approach were identical to those obtained using the Bayesian analysis. As shown in the phylogenetic tree (Figure 1), the mt genome reported here was clustered in genus *Tringa*, including *Tringa erythropus* (GenBank accession number: KX230491.1) and *Tringa ochropus* (GenBank accession number: KX668223.1).
